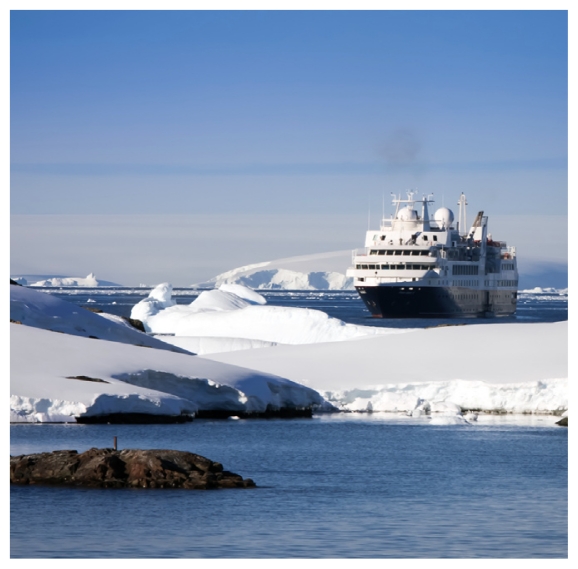# The Beat

**Published:** 2010-12

**Authors:** Erin E. Dooley

## Clearer Picture of Ground-Level Ozone Formation

Knowing how hydroxyl radicals combine with nitrogen dioxide from fossil fuel burning is important for better predicting the formation of air pollutants such as ground-level ozone and nitric acid. Now scientists have filled in some important knowledge gaps about this chemical reaction: the overall speed at which hydroxyl radicals and nitrogen dioxide react in given atmospheric conditions and the ratio of stable nitric acid to unstable nitric acid that is formed under such conditions.^1^ Their findings suggest most current computer models may underestimate ozone levels by 5–10% in highly polluted areas.

**Figure:**
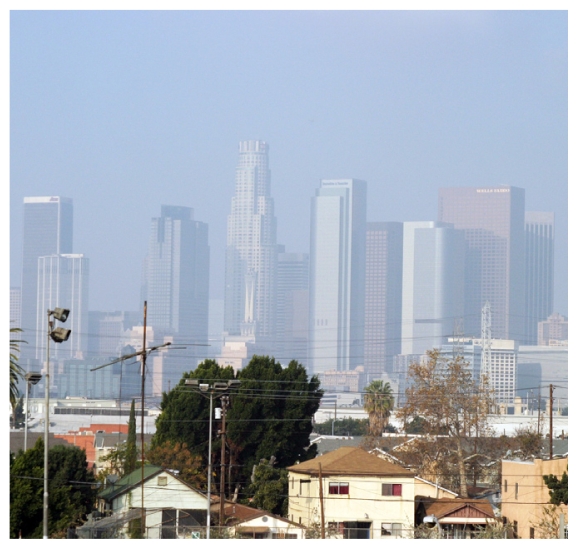


## Nature Rx

Over the next two years, the National Environmental Education Foundation’s Children and Nature Initiative expects to provide more than 1,200 health care providers with science-based knowledge, technical support, patient resources, and other tools they can use to prescribe outdoor time for children and their families.^2^ The goal of the program is to avail children of the physical and mental health benefits associated with unstructured outdoor play.^3^ Working with a number of federal and nonprofit partners, the program also will educate health care providers about safe, accessible local outdoor sites they can recommend to their patients.

**Figure:**
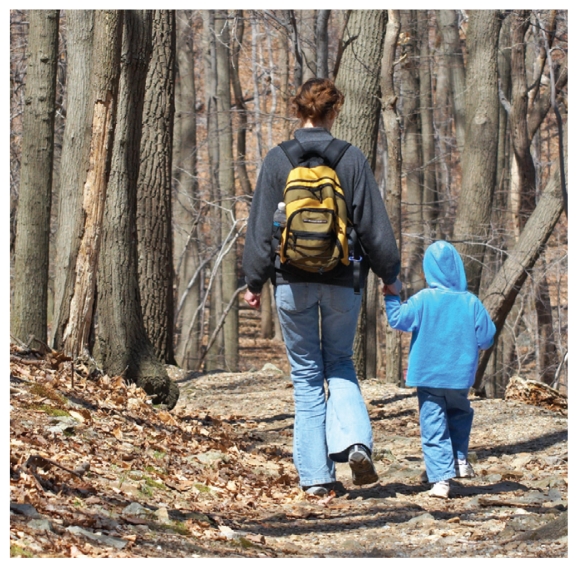


## Metrics for Partnerships

The NIEHS Division of Extramural Research and Training has issued the first draft of the Partnerships for Environmental Public Health (PEPH) Evaluation Metrics Manual for measuring partnership-building activities.^4^ The manual provides metrics that can be used to assess activities’ effectiveness and impact, evaluate program successes and challenges, justify further funding, and identify new audiences and applications for projects. Although designed for the NIEHS’s PEPH grantees, the manual applies to anyone working to build partnerships to address public health issues. The NIEHS invites the public to review the manual and provide feedback.^4^

## Health Care Policy through the Lens of Environmental Health

In September 2010 the Research Triangle Environmental Health Collaborative held a summit titled America’s Health Care Policy through the Lens of Environmental Health.^5^ Discussions in the realms of policies; research and analytical tools; and outreach, education, and mobilization all concluded that safeguarding public health requires a focus not just on health care reform but on reforming the public’s ideas about health itself. Collaborative leaders are preparing recommendations to present to Congress sometime in 2011.

## Report Highlights Arctic Issues

Several projections estimate the Arctic Ocean will be ice-free in the late summer months by the late 2030s—a dramatic ecologic change for this region. A new report prepared for Congress surveys potential issues that might arise as a consequence of diminishment of Arctic ice.^6^ The report outlines predictions of probable increases in shipping, fishing, tourism, and petroleum extraction activities in the region, potentially leading to increased pollution, stresses on wildlife and marine stocks, and impacts on traditional livelihoods. It also touches on concerns about potential health problems related to climate change that may particularly affect Arctic indigenous peoples.

**Figure:**